# Which resistance training is safest to practice? A systematic review

**DOI:** 10.1186/s13018-023-03781-x

**Published:** 2023-04-12

**Authors:** Thiago Teixeira Serafim, Eliton Stanley de Oliveira, Nicola Maffulli, Filippo Migliorini, Rodrigo Okubo

**Affiliations:** 1 Physiotherapy Nucleus Orthopedic Trauma of Health and Sports Science of the Santa Catarina State (UDESC), Florianópolis, Brazil; 2 Department of Medicine, Surgery and Dentistry, University of Salerno, 84081 Baronissi, SA Italy; 3 School of Pharmacy and Bioengineering, Keele University Faculty of Medicine, Stoke on Trent, ST4 7QB England; 4 Queen Mary University of London, Barts and the London School of Medicine and Dentistry, Centre for Sports and Exercise Medicine, Mile End Hospital, London, E1 4DG England; 5 Department of Orthopaedic, Trauma, and Reconstructive Surgery, RWTH University Hospital, Pauwelsstraße 30, 52074 Aachen, Germany

**Keywords:** Resistance training, Injury, Strength training, High-intensity functional training, Weightlifting

## Abstract

**Background:**

The combination of resistance training (RT) and aerobic training is believed to achieve the best effects. Several different aerobic training methods have emerged in combination with or as a substitute for traditional RT. This study wished to verify which RT is safest in terms of injury prevalence and incidence. Also, it ascertained the characteristics of the injured subjects, the level of severity of the injuries and what definitions of injuries the available studies use.

**Methods:**

This systematic review followed the PRISMA recommendations and was registered in PROSPERO with the number CRD42021257010. The searches were performed in the PubMed, Cochrane and Web of Science, electronic databases using the Medical Subject Headings terms "Resistance training" or "Strength training" or "Crossfit" or “Weightlifting” or “Powerlifting” combined (AND) with "Injury" or "Injuries" or "Sprain" AND “Incidence” or “Prevalence” AND “Epidemiology” or “Epidemiological” in the title or abstract. The last search was performed on March 2023. To be included in the review, the studies had to be available as full text, be clinical trials focusing on epidemiological injuries of resistance training. There was no time limit for the selection of articles. To assess the quality of the studies, the Strengthening the Reporting of Observational Studies in Epidemiology (STROBE) was used.

**Results:**

The initial literature search resulted in 4982 studies. After reading the titles, abstracts and full text, 28 articles were selected for data extraction. Seventeen investigated the injuries in HIFT/CrossFit, three in powerlifting, three in strength training, three in weightlifting and one in strongman. In addition, one study examined the HIFT/CrossFit and weightlifting. The incidence of injuries presented in the studies ranged from 0.21/1000 h to 18.9/1000 h and the prevalence of injuries was 10% to 82%. In the quality assessment for STROBE, five studies were classified at level A, 21 at level B and two at level C.

**Conclusion:**

This systematic review showed that traditional strength training is the safest RT method, and strongman is the least safe regarding injuries. Few studies have been rated highly according to STROBE. Furthermore, few studies have been published on some RT methods. These two factors make it difficult to generalize the results.

**Supplementary Information:**

The online version contains supplementary material available at 10.1186/s13018-023-03781-x.

## Background

The combination of resistance training (RT) associated with aerobic training is ideal for the best performance [1–3]. With the growth of such information and the encouragement for the greater practice of physical exercise, different RT methods have emerged [4–6]. Within these modalities, when considering health, well-being and quality of life, there was less concern only with aesthetics or performance gains within the sport [1, 2]. Therefore, studies that evaluate variables related to exercise safety are important [7]. Studies on the incidence and prevalence of injuries are important to identify risk factors within the modality and develop preventive strategies [8, 9]. The comparison between one modality and another is also important for practitioners to choose the best and safest RT method. This study wished to verify which RT is safest in terms of injury prevalence and incidence. Also, it ascertained the characteristics of the injured subjects, the level of severity of the injuries and what definitions of injuries the available studies use.

## Methods

### Protocol registration

This systematic review followed the Preferred Reporting Items for Systematic Reviews and Meta-Analyses (PRISMA) recommendations [10] and was registered in PROSPERO (ID CRD42021257010). The searches were performed in the PubMed, SPORTDiscuss and Web of Science, electronic databases using the following keywords (Additional file 1): "Resistance training" or "Strength training" or "CrossFit" or “Weightlifting” OR “Powerlifting” AND "Injury" or "Injuries" or "Sprain" AND “Incidence” or “Prevalence” AND “Epidemiology” or “Epidemiological”. The last update of the database search was conducted on March 2023.

### Eligibility criteria

Studies were deemed eligible according to the PICOS criteria [10, 11] (Table 1). To be included in the review, the studies had to be available as full text, and be clinical trials focusing on epidemiological aspects of injuries that occurred with RT. There was no time limit for the selection of articles. Literature reviews, case reports, editorials, letters to the editor, technical notes and articles published in languages other than English were excluded.

**Table 1:** PICOS framework

Criteria	Inclusion	Exclusion
P	Adults	< 18 years old
I	Weightlifting, powerlifting, Crossfit, HIFT, strongman, traditional strength training, bodybuilding	HIIT, calisthenics, military training, gymnastics
C	Others physical activity	–
O	Injury prevalence and/or incidence	–
S	Observational or clinical trials	Literature reviews, case reports, editorials, letters to the editor, technical notes

*HIFT* high-intensity functional training, *HIIT* high-intensity interval training

### Selection of studies and data extraction

The studies were independently screened by two reviewers (TTS and ESO) for inclusion. Each reviewer studied the title of each article identified through the search, followed by examination of the abstracts. Subsequently, the full text of the articles which passed the previous stages was analysed. Disagreements between reviewers were resolved by a third senior reviewer experienced in systematic reviews and meta-analyses (RO).

### Data extraction

The data collected by two authors (TTS and ESO) from the articles referred to the sample size, type of resistance training, incidence and prevalence of injuries, associated factors, severity, and definition of injuries. The American College of Sports Medicine (ACSM) defines resistance training for health and fitness as “a form of physical activity that is designed to improve muscle fitness by exercising a muscle or muscle group against external resistance” [12]. Resistance or strength training is widely performed in contemporary health and fitness environments through the use of equipment such as free weights, sectorized weight machines, plate loaded machines, weighted balls, resistance bands, and body weight resistance equipment [13].

### Quality assessment

To assess the quality of the studies, the Strengthening the Reporting of Observational Studies in Epidemiology (STROBE) was used. The scale has a checklist with 22 items that receive scores from 0 (does not meet) to 1 (complies). Depending on the sum of items reached by the study [14, 15] when the study fulfilled more than 80% of the criteria established in the STROBE, the study is considered high quality if 50% to 80% of the STROBE criteria are met [16]. On the other hand, if the considered study met less than 50% of the STROBE criteria, low quality if detected [16].

## Results

### Search results

**Fig. 1:**
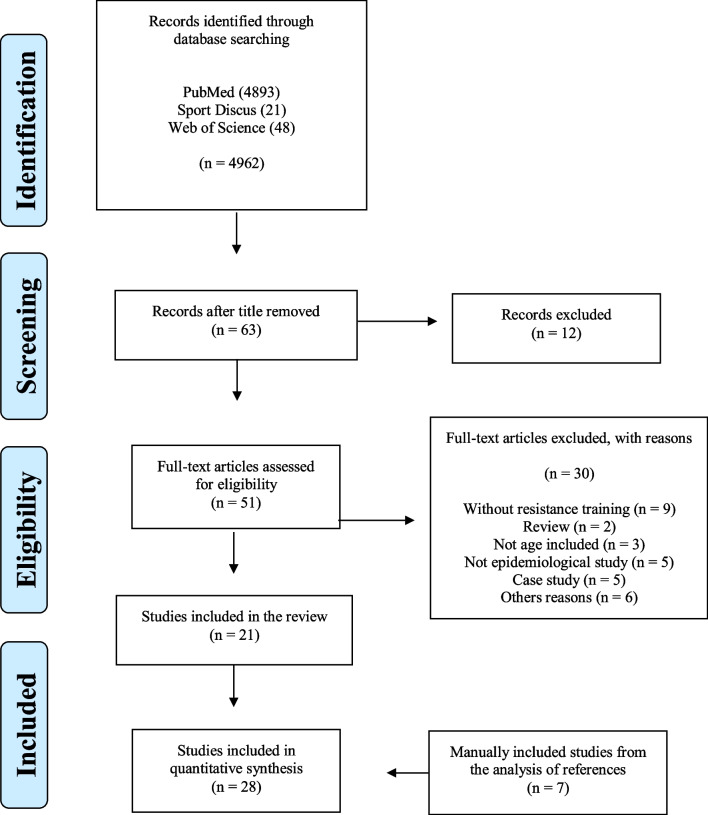
Flow chart of the literature search

The initial literature search resulted in 4982 studies. After reading the titles, 4899 were excluded, leaving 63 for evaluating the abstracts. Twelve studies were excluded at this stage, leaving 51 for further evaluation. 21 were selected for data extraction. Seven investigations were selected searching the references by hand. Eventually, 28 articles were selected for data extraction (Fig. 1).

### Patient demographic

Data from 13.127 RT practitioners were collected. The mean age was 28.7 ± 6.4 years. Their average weekly training was 2 to 6.10 workouts per week. The generalities and patient demographic of the included studies is shown in Table 2.

**Table 2:** Generalities and patient demographic of the included studies

Author and year	*N* (M; W)	Age	RT	Training/week	Injury incidence/1000 h	Injury prevalence
Moran et al. [27]	117 (66; 51)	35.10	HIFT/Crossfit	–	2.10	12.80%
Larsen et al. [32]	168 (51; 117)	29.20	HIFT/Crossfit	–	2.66	13.10%
Feito et al. [20]	3049 (1566; 1483)	37.30	HIFT/Crossfit	–	0.21	16.00%
Weisenthal et al. [61]	381 (231; 150)	–	HIFT/Crossfit	–	–	22.00%
Montalvo et al. [22]	191 (94; 97)	31.69	HIFT/Crossfit	4.39	2.3	26.18%
Feito et al. [17]	3049 (1566; 1483)	36.80	HIFT/Crossfit	–	0.74	30.50%
Szeles et al. [18]	406 (198; 208)	32.10	HIFT/Crossfit	3.90	18.90	32.80%
Alekseyev et al. [62]	885 (589; 296)	29.00	HIFT/Crossfit	–	–	33.30%
Aune and Powers [25]	247 (142; 105)	38.90	HIFT/Crossfit	3.50	2.71	34.00%
Teixeira et al. [44]	213 (112; 101)	29.74	HIFT/Crossfit	–	7.10	38.50%
Toledo et al. [63]	184 (97; 87)	28.70	HIFT/Crossfit	4.60	3.30	38.60%
Tafuri et al. [55]	454 (325; 129)	28.80	HIFT/Crossfit	4.00	–	39.90%
Cheng et aal [64]	244 (117; 127)	33.20	HIFT/Crossfit	–	–	46.00%
Escalante et al. [65]	159 (88; 71)	31.30	HIFT/Crossfit	4.30	3.30	46.50%
Mehrab et al. [66]	449 (266; 183)	31.90	HIFT/Crossfit	3.90	–	56.10%
Tawfik et al. [30]	270 (137; 132)	34.00	HIFT/Crossfit	–	–	62.20%
Hak et al. [45]	132 (93; 39)	32.20	HIFT/Crossfit	–	3.10	73.50%
Elkin et al. [33]	411–122 CrossFit; 289 weightlifting (–)	HIFT/CrossFit (37.45); weightlifting (31.62)	HiFT/Crossfit and Weightlifting	4.40 (HIFT/Crossfit); 4.50 (Weightlifting)	–	60.67% (HIFT/CrossFit); 46.71% (weightlifting)
Siewe et al. [56]	245 (219; 26)	37.80	Powerlifting	–	–	43.30%
Strömbäck et al. [39]	104 (–)	28.30	Powerlifting	3.60	–	70.00%
Keogh et al. [40]	101 (82; 19)	36.60	Powerlifting	6.10	4.00	–
Surakka et al. [19]	226 (83; 143)	44.00	Strength training	2.00	–	10.00%
Little et al. [67]	167 (63; 104)	69.00	Strength training	2.00	–	13.80%
Kim et al. [68]	210 (125; 85)	–	Strength training	–	–	14.00%
Winwood et al. [52]	213 (213; 0)	31.70	Strongman	–	5.50	82.00%
Junge et al. [69]	255 (–)	–	Weightlifting	–	–	16.90%
Calhoon et al. [57]	873 (–)	–	Weightlifting	–	3.30	–
Raske and Norlin [70]	135 (–)	30.10	Weightlifting	–	2.70	48–76%

*M* men, *W* woman, *HIFT* high-intensity interval training, *RT* resistance training

Seventeen studies evaluated the number of injuries in HIFT/CrossFit, three in powerlifting, three in strength training, three in weightlifting, and one in Strongman. In addition, one study looked at HIFT/CrossFit and weightlifting. Overall, the incidence of injuries ranged from 0.21/1000 h to 18.9/1000 h [17, 18] and the prevalence of injuries was 10% to 82% [19, 20]. Within the HIFT/CrossFit, the mean injury was 4.2/1000 h and 52.5%, respectively. In powerlifting, the mean prevalence of injuries was 56.6% and the incidence of 4/1000 h. Strength training studies did not show the incidence of injuries, with a mean prevalence of 12.6%. The only study on strongman reported an injury incidence of 5.5/1000 h and an injury prevalence of 82%. Weightlifting practitioners had 3.2/1000 h of injury incidence and 46.2% of injury prevalence, respectively. The greatest number of injuries were located in the shoulders [21–26], followed by the back [27–29]. Some studies analysed factors associated with injuries, as well as their severity and cause. Of the 28 studies included, 21 had explicitly defined an “injury” in their methods (Table 3).

**Table 3:** Injury characteristics

Author and year	Injury	Associated factors	Severity	Injury definition
Moran et al. [27]	Back 33,3% Knee 20% Wrist 13.3%	Male sex (+); Previous injury (+); Asymmetry on the FMS (+); Previous experience of Olympic lifting and/or gymnastics (−); Coaching exposure (−); Age (−); BMI (−)	15 ± 24 days	Any physical complaint that was sustained during CrossFit training that resulted in a participant being unable to take a full part in future CrossFit training (i.e. a “time-loss” definition)
Larsen et al. [32]	Back 25% Knee 21.4% Elbow/Hand 17.9%	Sex (−); Age (−); BMI (−); Exercise prior HIFT/Crossfit (−)	–	Pain, soreness, stiffness or swelling
Feito et al. [20]	–	< 3 days/week more injuries (+); < 6 months experience training (+); Age (−); Sex (−)	–	Any muscle, tendon, bone, joint, or ligament injury sustained while doing CrossFit that resulted in your consultation with a physician, or healthcare provider AND caused you to stop or reduce your usual physical activity, your typical participation in CrossFit, or caused you to have surgery
Weisenthal et al. [61]	Shoulder 25% Back14.2% Knee 13%	Not supervision (+); Male sex (+);	–	Any new musculoskeletal pain, feeling, or injury that results from a CrossFit workout and leads to 1 or more of the following options: Total removal from CrossFit training and other outside routine physical activities for > 1 week; Modification of normal training activities in duration, intensity, or mode for > 2 weeks; Any physical complaint severe enough to warrant a visit to a health professional
Montalvo et al. [22]	Shoulder 22.6% Knee 16.1% Back 12.9%	Competitor (+); Physical active outside Crossfit (+); Sex (−); Fitness level before Crossfit (−); Warm up (−); Cool down (−)	–	Any physical damage to a body part that caused them to miss or modify one or more training sessions or hindered activities of daily living
Feito et al. [17]	Shoulders 39% Back 36% Knees 15%	> 3 years experience training (+); Male sex (+)	–	Any muscle, tendon, bone, joint, or ligament injury sustained while doing CrossFit that resulted in your consultation with a physician, or health care provider, AND caused you to stop or reduce your usual physical activity, your typical participation in CrossFit, or caused you to have surgery
Szeles et al. [18]	Shoulders 19% Back 15% Knees 11.7%	Previous injury (+); Quality of movement (+); Alternating Rx/scaled (+); Protective equipment (−); Stretching exercises (−); Sex (−); Preventive exercises (−); Practice of other sports (−)		Injury is defined as any musculoskeletal injury or pain (in joints, bones, ligaments, tendons, or muscles) that prevented an athlete from exercising for at least 1 day
Alekseyev et al. [62]	Back 32.2% Shoulder 20.79% Knee 17%	Sex (−); > 1 year experience training (+); > 9 h training/week; Stretch before exercise (+)	–	–
Aune and Powers [25]	Shoulders 15% Back 12% Knees 12%	–	–	–
Teixeira et al. [44]	Shoulder 36.6% Back 19.5% Knee 12.2%	Competitor national level (+); > 2 years practice time (+); Sex (−); Objective (−); Exercise before Crossfit (−); Physical active outside Crossfit (−); Category (−)	−	Any pain or injury that has impaired the life routine or modified the participant’s training sessions
Toledo et al. [63]	Shoulder Wrist Ankle	Sex (−); Experience training (+); < 3 training/week for women (+)		Any damage sustained during training that prevented the participant from training, working, or competing in any way and for any period
Tafuri et al. [55]		Experience time (+); Clean ceiling (+); Previous injury (+); On-ramp course (+)	22.6 ± 31 days	
Cheng et al. [64]	Back 88% Shoulder 84% Wrist 62%	Previous injury (+); Sex (−); nonaffiliated (+); Experience training (−); Fitness level (−); BMI (−); Age (−)	–	An injury was defined as a new musculoskeletal pain, sensation, or discomfort that resulted in any of the following26: Total removal from CrossFit training and other outside routine physical activities for > 1 week; Modification of normal training activities in duration, intensity, or mode for > 2 weeks; Any physical complaint severe enough to warrant a visit to a health professional
Escalante et al. [65]	Back 18.1% Knee 12.5% Wrist 10.2%	–	–	An injury that met one of the following criteria within the last 12 months of CrossFit® participation: required the individual to seek a healthcare professional to diagnose/treat the injury; modification of normal training activities for more than two weeks; total removal from CrossFit® and other physical activity for more than one week; or any injury that required loss of time from employment. The survey also asked about injury location as well as the diagnosis (if applicable), severity, time lost from training, and history of a related injury
Mehrab et al. [66]	Shoulders 28.7% Back 15.8% Knees 8.3%	< 6 months experience training (+); Sex (−); BMI (−); Participation in other sports (−); Warmup (−)	–	Any new musculoskeletal pain, feeling, or discomfort as a result of a CrossFit workout that met 1 of the following criteria: Total removal from CrossFit training and other outside routine physical activities for > 1 week; Modification of normal training activities in duration, intensity, or mode for > 2 weeks; Any physical complaint severe enough to warrant a visit to a health professional
Tawfik et al. [30]	–	–	–	A CrossFit related injury was defined as any of the following which occurred as the result of CrossFit training: (1) inability to train for greater than one week; (2) needing to modify training duration, activity, or intensity for greater than two weeks; (3) any complaint that led to a doctor visit
Hak et al. [45]	–	–	–	–
Elkin et al. [33]	Shoulder 46.41% Back 38.28% Hip 9.09%			–
Siewe et al. [56]	Shoulder 16.3% Back 15.1%; Lower extremity 13.9%	–	Most injuries had a mild (39%) to moderate (39%) effect (severity) on training, meaning that the lifters only had to make relatively minor modifications to the prescribe	An incident leading to an interruption in training or competition. The fourth part focused on general disorders, and finally the fifth part assessed parameters regarding life style, nutrition, and medical therapy
Strömbäck et al. [39]	Lobopelvic area 31.5% Hip 27.4% Shoulder 26%	Personal best in the deadlift (+); alcohol ingestion (+); Male sex (+); Frequency training (−)	–	A condition of pain or impairment of bodily function that affected powerlifters’ training
Keogh et al. [40]	Shoulder 36% Back 24%	–	–	Any physical damage to the body that caused the lifter to miss or modify one or more training sessions or to miss a competition
Surakka et al. [19]	Thigh 37% Ankle 19% Knee 19%	–	–	–
Little et al. [67]	–			Injury is defined as a self–reported muscle, tendon, bone, ligament, or joint injury?
Kim et al. [68]	–	–	–	–
Winwood et al. [52]	Back 24% Shoulder 21% Biceps 11%	< 30 years old (+); > 105 kg (+)	Moderate 47%; Mild 33%; Major 20%	Any “physical damage to the body that caused the strongman athlete to miss or modify one or more training sessions or miss a competition”
Junge et al. [69]	–	–	–	Any musculoskeletal complaint (traumatic and overuse) newly incurred due to competition and/or training during the XXIXth Olympiad in Beijing that received medical attention regardless of the consequences with respect to absence from competition or training
Calhoon et al. [57]	Back Shoulder	–	< 7 days	Injury classifications were acute, chronic, recurrent, or complication. Acute injuries are "injuries with rapid onset due to a traumatic episode, but with short duration."6 A chronic injury is "an injury with long onset and duration."6 A recurring injury involves recovery and reinjury for a particular condition
Raske and Norlin [70]	Shoulder Back	–	–	An inability to train or compete as planned because of symptoms

*BMI* body max index, *HIFT* high-intensity functional training

### Quality assessment

The studies were evaluated using the STROBE Checklist. The range of points acquired by the studies on the scale ranged from 8 [30] to 19 [31–33]. Five studies were classified at level A, 21 at level B and two at level C.

## Discussion

This study investigates the injury rate among resistance training partitioners. Traditional strength training showed a lower injury rate, unlike Strongman, which was the RT method with the highest injury rate in the selected studies. In general, the reported injuries are of high severity, with shoulders and back being the most commonly affected anatomical areas. The injuries definitions were different between the selected studies.

Within sports, there is a particularity that makes it difficult to characterize an injury. Sport, unlike other contexts, makes the athlete or practitioner continue their training or participate in some competition even with pain or loss of function. Therefore, the simple absence from training or competition cannot always be characterized as an injury [34, 35]. With this in mind, most selected studies characterised the injury as any pain or change in performance within the training modality and exercises performed. Other studies were less stringent, and only considered injuries when the subject did not practice for some time. This agrees with the definition of a sport injury as a pathological process that interrupts training or competition and can lead the athlete to seek medical treatment [36]. There is a perceived difficulty in standardizing the definition of injuries in studies. No matter how difficult it may be, this must include within its definition the inability to perform the sport [34].

Traditional ST presented the lowest prevalence of injuries, at an average of 13%, demonstrating the safety of the practice of traditional ST. The low incidence (< 1/1000 h) indicated the safety of the practice [4]. The safety of traditional ST can also be explained by the different profiles of the training method [37, 38]. While other RT modalities put a greater focus on the task and constant challenge related to performing complex movements at higher intensities, traditional ST mostly focuses on specific muscle contraction [38]. Powerlifting had a low incidence of injury, very similar to HIFT/CrossFit and weightlifting [39, 40]. Powerlifting usually occurs from the high loads used in deadlift, squat and bench press [41]. Using high loads requires excellent technique and reduces the chances of injuries [42, 43]. Most of the studies identified on HIFT/CrossFit, with an average of 4.22 lesions per each 1000 h of exposure. Even with a low average, two studies showed a high rate of injury incidence [18, 44]. Szeles et al. evidenced an incidence of 18.9/1000 h lesions, well above the others [18]. This difference of almost 5 times the mean value can be explained by the different methods used to define an injury. The main justification is the non-standardization of the definition of injury. In this review, for example, seven studies had no definition of injury. Furthermore, many studies have different definitions, which increases the subjectivity of the interpretation [4]. Hak et al. found almost double the prevalence of injuries [45]. In one of the first epidemiological studies of HIFT/Crossfit conducted online, the online questionnaire, depending on how it is disseminated, may be biased towards the target audience of the survey [46, 47], as a study of injuries in a sport can draw more attention to subjects who have already had an injury. Studies with higher injury prevalence often define injuries as any pain or loss of function that makes the subject change training or results in a reduction in training performance. Other studies with lower rates have less stringent definitions with a lower degree of rigidity or no definition at all. This further increases the importance of standardizing the method of studies [48, 49].

The injury rate in weightlifting is similar to HIFT/CrossFit. However, a smaller number of studies were found, which makes it more difficult to consider fewer results as accurate as the HIFT/CrossFit. A systematic review showed values similar to those of this review [50]. The severity of injuries in this study varied greatly, and this may occur because some accidents take place during training [50, 51]. The highest prevalence found in the studies was that of Strongman [52], a sport in which athletes perform with high loads and varied movements. Specific training is responsible for increasing the chances of injury by 1.9 times when compared to traditional ST [52].

Most injuries occurred in the shoulders, followed by the back. These results are in line with previous studies in HIFT/CrossFit, weightlifting and powerlifting [46, 50], given the high loads and large ranges of motion [50, 53, 54]. It is necessary to have good stability of the scapulothoracic complex to allow less overload on the glenohumeral joint. Lower trapezius and serratus anterior activation are critical in overhead movements [47]. A single training method altered the pattern of shoulder and back injuries. Only one of the traditional RT studies verified this and realized that injuries in the lower limbs probably occurred through running and jumping [19]. Most injuries were classified as moderate, but few studies included this variable in their results [27, 52, 55–57]. Furthermore, the small number of studies that verified the severity of injuries does not allow generalization of the results. As these sports do not involve a constant change of direction and physical contact, injuries tend to be less severe [47, 58, 59]. Most studies did not find an association between the practitioner's sex and the occurrence of injuries. Previous untreated injuries seem to predispose to new injuries. Some of these RT methods are recent, which makes their practitioners come from other sports with an injury already treated [47, 60]. Individuals who start practising HIFT/CrossFit are 3.75 times more likely to get injured in practice [60]. Athletes with previous shoulder injuries are eight times more likely to injure the area compared to athletes with healthy shoulders [25]. In the practical context, all RT methods seem safe. Strongman reported the highest rate of injuries, but only one study was included in the analysis.

## Conclusions

Traditional strength training is the safest RT method, and Strongman is the least safe regarding injuries. The anatomical sites with the highest rate of injuries are the shoulders and the lumbar region. Study methods need to be better standardized to prevent discrepant and heterogeneous results.

## Supplementary Information

**Additional file 1**. Search strategy.

## Data Availability

The data that support the findings of this study are available from Thiago Teixeira Serafim, but restrictions apply to the availability of these data, which were used under license for the current study, and so are not publicly available. Data are, however, available from the authors upon reasonable request and with permission of Thiago Teixeira Serafim via e-mail.

## Abbreviations

RT: Resistance training
STROBE: Strengthening the Reporting of Observational Studies in Epidemiology
PRISMA: Preferred Reporting Items for Systematic Reviews and Meta-Analyses
MeSH: Medical Subject Headings
ACSM: American College of Sports Medicine
HIFT: High-intensity functional training
ST: Strength training
