# Supplementary material for: Which resistance training is safest to practice? A systematic review
Source: J Orthop Surg Res. 2023 Apr 12;18:296. doi: 10.1186/s13018-023-03781-x (PMC10099898; doi:10.1186/s13018-023-03781-x)
Supplement: Supplementary file 1 — Additional file 1. Search strategy. [file 13018_2023_3781_MOESM1_ESM.docx]

**Supplementary material.** Search strategy

| **Order** | **Term** |
| --- | --- |
| **#1** | “Resistance training” |
| **#2** | Strength training” |
| **#3** | Crossfit |
| **#4** | Weightlifting |
| **#5** | Powerlifting |
| **#6** | Injury |
| **#7** | Injuries |
| **#8** | Sprain |
| **#9** | Incidence |
| **#10** | Prevalence |
| **#11** | Epidemiological |
| **#12** | Epidemioogy |
| **#13** | #1 OR #2 OR #3 OR #4 OR #5 |
| **#14** | #6 OR #7 OR #8 |
| **#15** | #9 OR #10 |
| **#16** | #11 OR #12 |
| **#17** | #13 AND #14 AND #15 AND #16 |
